# Expression, Purification, and Characterization of a Novel Hybrid Peptide with Potent Antibacterial Activity

**DOI:** 10.3390/molecules23061491

**Published:** 2018-06-20

**Authors:** Xubiao Wei, Rujuan Wu, Lulu Zhang, Baseer Ahmad, Dayong Si, Rijun Zhang

**Affiliations:** Laboratory of Feed Biotechnology, State Key Laboratory of Animal Nutrition, College of Animal Science and Technology, China Agricultural University, Beijing 100193, China; Weixubiao01@cau.edu.cn (X.W.); Lingxiabenpao@aliyun.com (R.W.); zhanglulu09@cau.edu.cn (L.Z.); dr.baseerahmadkhan@gmail.com (B.A.)

**Keywords:** hybrid peptide, fusion expression, *Escherichia coli*, antibacterial activity, stability

## Abstract

The hybrid peptide cecropin A (1–8)–LL37 (17–30) (C–L), derived from the sequence of cecropin A (C) and LL-37 (L), showed significantly increased antibacterial activity and minimized hemolytic activity than C and L alone. To obtain high-level production of C–L, the deoxyribonucleic acid sequence encoding C–L with preferred codons was cloned into pET-SUMO to construct a fusion expression vector, and overexpressed in *Escherichia coli* (*E. coli*) BL21 (DE3). The maximum fusion protein (92% purity) was obtained with the yield of 89.14 mg/L fermentation culture after purification with Ni-NTA Sepharose column. The hybrid C–L was cleaved from the fusion protein by SUMO-protease, and 17.54 mg/L pure active C–L was obtained. Furthermore, the purified C–L showed identical antibacterial and hemolytic activity to synthesized C–L. Stability analysis results exhibited that the activity of C–L changed little below 80 °C for 20 min, but when the temperature exceeded 80 °C, a significant decrease was observed. Varying the pH from 5.0 to 10.0 did not appear to influence the activity of C–L, however, pH below 4.0 decreased the antibacterial activity of C–L rapidly. Under the challenge of several proteases (pepsin, trypsin, and proteinase K), the functional activity of C–L was maintained over 50%. In summary, this study not only supplied an effective approach for high-level production of hybrid peptide C–L, but paved the way for its further exploration in controlling infectious diseases of farm animals or even humans.

## 1. Introduction

In recent years, more and more efforts have been paid on developing new safe therapeutic compounds to cope with the increasing microbial resistance to conventional antibiotics [1,2]. Antimicrobial peptides (AMPs), a kind of naturally distributed short amphipathic cationic polypeptide, were key players of the defense systems for the majority of living organisms, by killing the invading pathogenic organisms directly and modulating the immune response [3,4,5]. Studies on both living organisms and simulated membranes have documented that the mechanism of AMPs is completely different from traditional antibiotics. AMPs exerted their antimicrobial activity through several models, including “pore-forming”, “barrel-stave”, “carpet” model, and so on, and were reported to be less prone to drug resistance than traditional antibiotics [6,7,8]. Therefore, AMPs show great potential as a pharmaceutical for the treatment of increasing antibiotic-resistant bacterial infections and immunological disease [2,3,4,5,6,7,8].

The human cathelicidin peptide, LL37 (L), plays a critical role in the process of antimicrobial infection and wound healing, but also exhibits undesirable hemolytic activity against host cells [9,10,11]. This property greatly limits its application in clinic. Cecropin A (C), isolated from *Hyalophora cecropia*, is a small linear helical peptide with broad bioactivities. It shows antibacterial, anti-inflammation and anticancer activities, and does not lyse eukaryotic cells [2,12,13]. Hybridizing different AMPs has been an effective method to obtain novel hybrid AMPs with elevated antibacterial activity but minimized cytotoxicity [14,15]. In the previous study from our laboratory, a novel hybrid peptide cecropin A (1–8)-LL37 (17–30) (C–L) was designed by combining the N-terminal amphipathic α-helix fragment of C with the core antimicrobial fragment of L. It possessed significantly greater antibacterial activity against a wide range of pathogens, but displayed minimized (nearly no) cytotoxicity compared to parental peptides (C and L) [2]. To obtain large quantities of C–L for physiological and structural investigations, effective production approaches are necessary.

*Escherichia coli* (*E. coli*) expression system is effective to produce proteins and peptides, due to its high production, relative simplicity of the DNA manipulation, and low cost [16,17,18,19]. By conjoining a partner to the N-terminal of AMPs, a fusion strategy has been employed to eliminate the toxicity of AMPs to host cell and protect AMPs from proteolytic degradation. There are various fusion partners [19,20,21,22], among which, SUMO was one of the most effective partners because not only does it facilitate the purification of the fusion protein with His-tag in N-terminal, but it can also be removed by SUMO protease specifically, allowing the recombinant proteins to retain the native N-terminus, which is a critical feature to the antimicrobial activity [20]. In the present research, SUMO–C–L was expressed in *E. coli*, recombinant C–L was purified, and its antibacterial and hemolytic activity and stability were also assayed.

## 2. Results

### 2.1. Construction of Expression Plasmids

**Figure 1:**
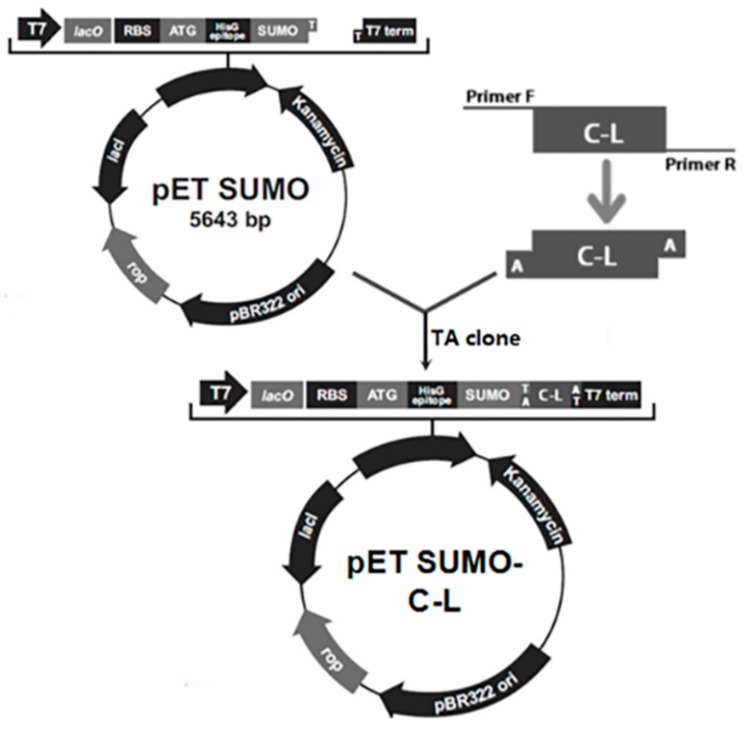
The schematic of construction of expression vector.

The schematic of construction of the expression vector was depicted in Figure 1. The C–L gene fragment was inserted downstream of SUMO gene to construct recombinant expression plasmids pET-SUMO–C–L. His-tag sequence was at the N-terminus of SUMO. The amino acid sequence of C–L was KWKLFKKIFKRIVQRIKDFLRN. The correct orientation of the insert was confirmed by PCR amplification and DNA sequencing (data not shown).

### 2.2. Expression of Fusion Protein SUMO–C–L

**Figure 2:**
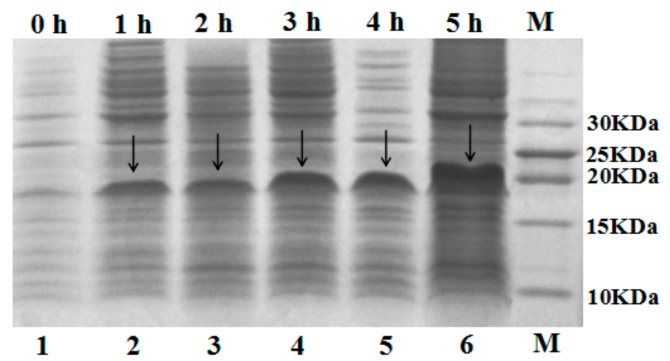
Tricine-SDS-PAGE analysis of supernatant with 1.5 mM IPTG induction. Lane 1, supernatant of non-induced *E. coli* BL21 (DE3); Lane 2, 3, 4, 5, 6 were supernatant of 1.5 mM IPTG-induced *E. coli* BL21 (DE3) for 1, 2, 3, 4, 5 h respectively; Lane M, marker. The arrows indicated fusion protein SUMO–C–L.

The recombinant plasmid pET-SUMO–C–L was transformed into *E. coli* BL21 (DE3) cells for expression. The gene of hybrid peptide C–L was expressed as a fusion protein in recombinant *E. coli* BL21 (DE3) cells with 1.5 mM IPTG induction. After cell disruption and centrifuge, the supernatant was analyzed by 16% tricine-SDS-PAGE. As shown in Figure 2, an apparent molecular mass of about 18 kDa was detected (calculated molecular mass, 18.1 kDa). After adding IPTG, the quantity of fusion protein SUMO–C–L increased, and the maximum was observed at 5 h, constituting up to 60.3% of all soluble proteins in the supernatant determined by Bandscan 5.0 software (Glyko, Novato, CA, USA).

### 2.3. Purification and Cleavage of Fusion Protein SUMO–C–L

**Figure 3:**
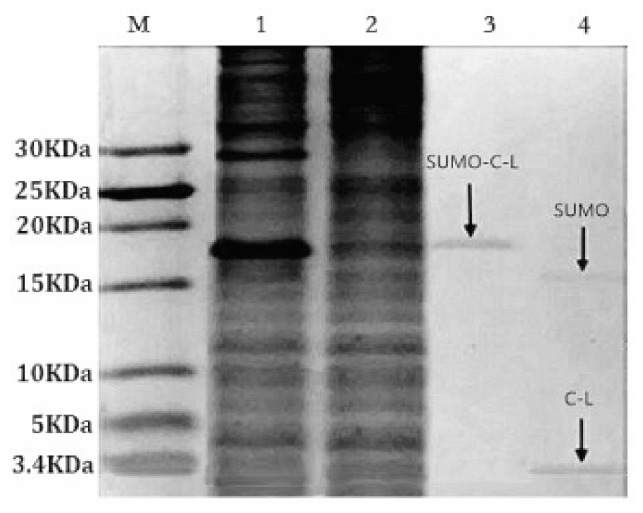
Tricine-SDS-PAGE analysis of purified fusion protein SUMO–C–L. Lane M, the molecular weight of marker; Lane 1, the supernatant of IPTG-induced *E. coli* BL21 (DE3); Lane 2, the supernatant of non-induced bacterial lysate; Lane 3, the purified fusion protein SUMO–C–L, arrow in the lane indicated the fusion protein SUMO–C–L; Lane 4, the enzymatic reaction solution of fusion protein, arrows in the lane were SUMO and recombinant C–L.

The soluble fusion protein SUMO–C–L with His-tag enabled the affinity purification through Ni-NTA Sepharose affinity column. Tricine-SDS-PAGE staining with Coomassie blue (Figure 3, lane 3) showed that the SUMO–C–L was purified to 92% (Bandscan 5.0 software, Glyko, Novato, CA, USA). The yield of purified fusion protein SUMO–C–L was approximately 89.14 mg/L fermentation culture. SUMO-protease recognizing and specifically cleaving the C-terminal Gly–Gly residues of the SUMO part was applied to release C–L sequence from the fusion protein. The recombinant C–L was cleaved from SUMO–C–L by enzymatic digestion with SUMO-protease at 30 °C for 3 h (Figure 3, lane 4). Two protein bands was observed in the lane 4, one was approximately 15 kDa, corresponding to the molecular weight of SUMO partner (calculated molecular mass, 15.2 kDa), and another one was 3 kDa, corresponding to the molecular weight of C–L (calculated molecular mass, 2.9 kDa).

### 2.4. Purification of Recombinant C–L

**Figure 4:**
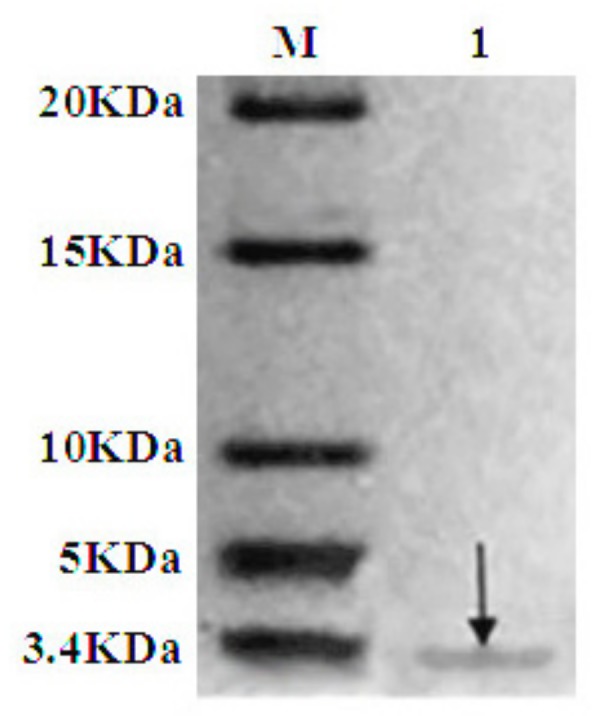
Tricine-SDS-PAGE and antibacterial analysis of purified recombinant C–L. Lane M, the molecular weight of marker; Lane 1, the purified recombinant C–L, arrow in the lane indicated the recombinant C–L.

**Figure 5:**
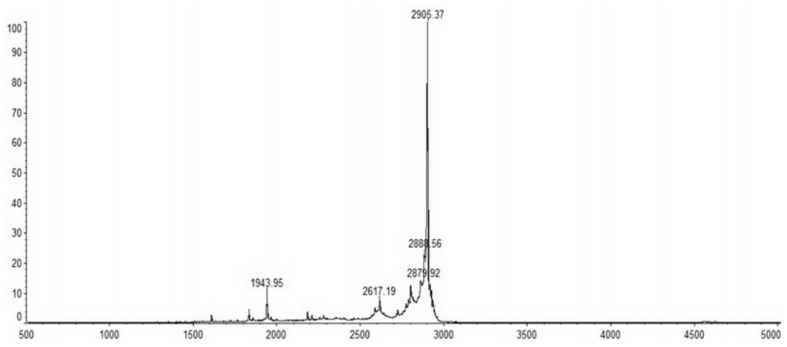
MALDI-TOF/TOF MS mass spectra of purified C–L.

**Figure 6:**
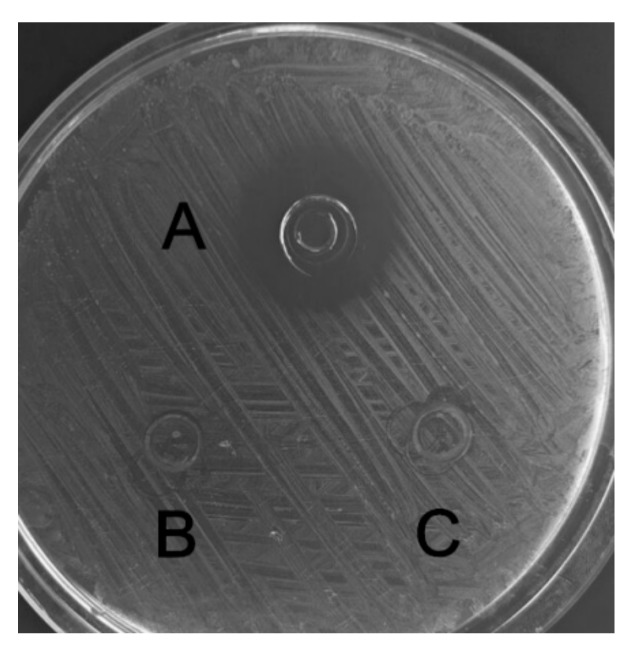
The antimicrobial activity of recombinant C–L against *S. aureus* ATCC25923. A, recombinant C–L (2 μg); B, fusion SUMO–C–L (10 μg); C, the negative control, sodium phosphate buffer (PBS).

The recombinant C–L was purified from the cleavage solution with Ni-NTA Sepharose affinity column. Approximately 17.84 mg/L recombinant C–L was collected. As shown in Figure 4, one single protein band (lane 1) with a molecular mass of about 3 kDa was observed in the dialysis liquid. To be notable, the exact molecular weight of C–L was 2905.37 Da determined by MALDI-TOF (Figure 5), which was basically consistent with calculated value of 2905.70. After incubation at 37 °C overnight, growth inhibition of (*Staphylococcus aureus*) *S. aureus* ATCC25923 was found around the cylinder to which had been added recombinant C–L, while no antibacterial activities of SUMO–C–L and sodium phosphate buffer (PBS) were observed (Figure 6).

### 2.5. Antibacterial and Hemolytic Activity of C, L, and C–L

The antibacterial and hemolytic activity (HC50) of C, L, and C–L was determined through microbroth dilution method (Table 1). When compared to parental peptides, the antibacterial activity of C–L was greatly improved. It showed that all the three indicator strains were more susceptible to C–L (MICs ranged 2.0–7.2 µg/mL) than L (MICs ranged 13.4–85.2 µg/mL) and C (MICs ranged 4.2–198 µg/mL) (*p* < 0.01). Only very low hemolytic activities of C–L to sheep erythrocyte cells were observed in this test (HC_50_ of recombinant and synthesized C–L was 221 and 219 µg/mL, HC_50_ >128 µg/mL was determined as no cytotoxicity) while the HC_50_ of C and L was 169 and 32 µg/mL, respectively (*p* < 0.01). Additionally, recombinant C–L exhibited similar antibacterial activity to synthesized C–L against *E. coli* CVCC245, *S. aureus* ATCC25923, and *Listeria monocytogenes* CVCC1599 (*p* > 0.05). The HC_50_ of recombinant C–L to sheep erythrocyte cells was agreed with that of synthesized C–L (*p* > 0.05). It is important that antibacterial display little or no hemolytic activity for their safe application in internal medicine.

### 2.6. Effect of Temperature, pH and Proteinases on C–L Activity

**Figure 7:**
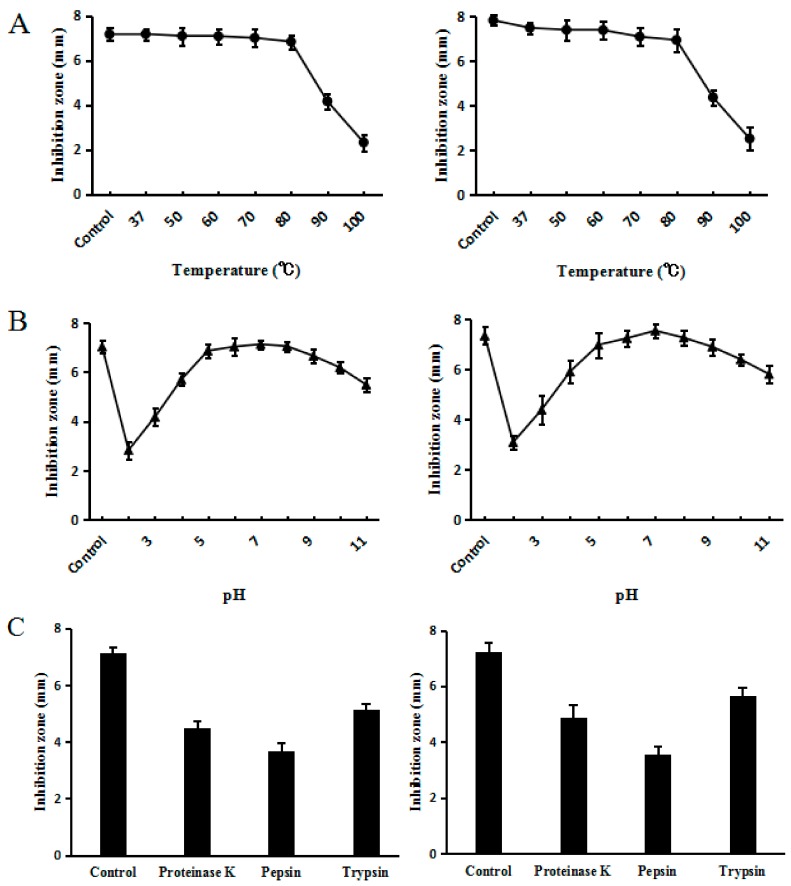
Effects of temperature (**A**), pH (**B**), and proteases (**C**) on recombinant (**left**) and synthesized C–L (**right**). (**A**) The peptide sample kept at 4 °C was used as a control. (**B**) The sample kept in the original culture (pH 6.0) was used as a control. (**C**) The original sample without any enzymatic treatment was used as a control. The graphs were derived from average values for three replicate experiments and error bars show standard deviations. *E. coli* ATCC25923 was used as the indicator strain.

If an application for humans or livestock is to be found for C–L, it is important to test for interference factors against its activity. Thermal stability is of great importance because medicine or feed products underwent several heat treatments before reaching the market. Other factors that could interfere with activity would be pH and enzyme stabilities, since peptides encounter different pH and enzymes at different digestion stages. Accordingly, the effects of temperature, pH, and proteolytic enzymes on the activity of C–L against *S. sureus* ATCC25923 were determined by inhibition zone assays. The results of heat stability assay confirmed that synthesized C–L was heat stable, as the antimicrobial activity of C–L was fully retained even after exposing it to 80 °C for 20 min; however, temperature above 80 °C reduced the activity of synthesized C–L significantly (Figure 7A). The residual activities of synthesized C–L were >80% over a broad pH range, from 4.0 to 11.0 (Figure 7B). Under the challenge of several proteases (proteinase K, trypsin, and pepsin), synthesized C–L maintained a portion of functional activity, as shown in Figure 7C. Additionally, compared with synthesized C–L, recombinant C–L was shown to retain almost the same stability when given the same heat, pH, and enzymes treatments as above.

## 3. Discussion

Hybridizing different AMPs has been a successful practice to improve the properties of native AMPs [14,15]. The hybrid peptide C–L, combining the core segment of L with the hydrophobic segment of C, was designed, and demonstrated excellent elevated antibacterial activity but minimized cytotoxicity, compared with C and L alone. To explore the pharmaceutical potential and medical importance of C–L, a cost-effective approach for large-scale production is required.

To date, various expression systems have been established for the production of recombinant AMPs [17,20,23]. Among them, fusion expression in *E. coli* system is a common strategy to produce AMPs, due to their cytotoxicity to host cells and sensitivity to proteases [24]. The expression vector pET-SUMO, which combines the pET plasmid with SUMO partner together, allows the target gene to be connected with SUMO by “TA” clone to express it effectively and stably under the control of strong T7 promoter [25]. Several AMPs have been efficiently produced using the SUMO fusing system in *E. coli* [16,26]. Thioredoxin, ubiquitin, and GST were also used as fusion partners in *E. coli* for AMP production [27,28,29]. The yield of thioredoxin-Mdc [28], ubiquitin-CA-MA2 [29], and GST-LL37 [27] in fermentation solution was 48, 32, and 8 mg/L, respectively. In the present study, the fusion protein SUMO–C–L was successfully expressed in *E. coli* BL21 (DE3) cells with the yield of 89.14 mg SUMO–C–L per liter fermentation liquid, which was extremely high, indicating the effectiveness of SUMO as a fusion partner.

It is worth noting that the soluble SUMO–C–L was not active, thus, it was necessary to release the recombinant C–L by enzymatic cleavage. SUMO protease had the ability to cut off the SUMO tag specifically, consequently allowing the recombinant C–L to retain the native N-terminus which was a critical feature for antimicrobial activity [30]. In the present study, the cleaved fusion protein was subjected to tricine-SDS-PAGE, and the size of recombinant C–L was consistent with the predicted molecular weight. After purification with Ni-NTA, the recombinant C–L was recovered at the yield of 17.34 mg/L, which was higher than 11.2 mg/L Mdmcec [28], 4.3 mg/L LL37 [16], and 7.9 mg/L cecropin B [31]. The purified recombinant C–L showed similar antimicrobial (higher than C and L) and hemolytic (lower than the parental peptides) activity to synthesized C–L, indicating that the hybrid C–L peptide with excellent antibacterial activity but no cytotoxicity (HC_50_ >128 μg/mL was determined as no cytotoxicity) was successfully expressed in *E. coli* BL21 (DE3) and purified with Ni-NTA Sepharose column.

The need to apply C–L in human or livestock prompted us to test for interference factors against its activity. The high thermal stability over a temperature range from 37 to 80 °C revealed the possibility of applying C–L in human or farm animals, where the body temperature in which the product must function is close to 40 °C for both poultry [32] and pigs [33]. More encouragingly, C–L maintained most of the activity in 80 °C, suggesting that it could withstand the high temperature to which the feed is exposed during pelleting.

Knowledge about the peptide function in the pH range of the gastrointestinal tracts is of crucial importance in the efficient application of an oral agent for animals. The pH in the stomach, small intestine, and large intestine of piglets 1 week postweaning was measured, and the values ranged from 1.6 to 4.4, 3.2 to 5.8, and 5.9 to 6.5, respectively [34]. In the present study, C–L showed significant activity in a wide pH range from 4.0 to 11.0. This feature enables C–L to realize its maximum potential in controlling pathogens, and in modulating the balance of gut flora within the pH range in the gastrointestinal tracts of recently weaned piglets. However, almost inevitably, a minor part of the C–L will lose its antibacterial activity due to gastric digestion, since the pH value in stomach is lower than 4.0, which could be ameliorated by some processing technologies, such as coating.

It is also important to assess the interference effects of gastric enzymes and pancreatic enzymes on C–L activity, such as pepsin and trypsin, respectively [35]. Additionally, another commonly used enzyme, proteinase K, was detected, which is not secreted in the gastrointestinal tracts of animals, but is useful for general digestion of proteins and removal of endogenous nucleases during the preparation of DNA and RNA. The results from the present study showed that C–L was, to some extent, resistant to proteolytic digestion by these enzymes. However, still nearly half reduction in activity was found when C–L was exposed to pepsin. A similar reduction in activity was also seen in Bovicin HC5 (a bacteriocin from *Streptococcus bovis* HC5) when it was processed by heat and proteinases treatments [36], which indicated the common problem of the susceptibility of antibacterial peptide activity.

## 4. Materials and Methods

### 4.1. Bacterial Strains, Plasmids, and Enzymes

Three pathogenic microorganisms were obtained from China Veterinary Culture Collection (CVCC, Beijing, China). They were *E. coli* CVCC245, *S. aureus* ATCC25923, and *Listeria monocytogenes* (*L. mono.*) CVCC1599. The plasmid pET-SUMO (Invitrogen, Carlsbad, CA, USA) was used as cloning vector. *E. coli* DH 5α and BL21 (DE3) (Novagen, Madison, WI, USA) were cultivated and selected in Luria-Bertani (LB) broth containing 50 μg/mL kanamycin at 37 °C, as the host for gene manipulation and expression of fusion protein, respectively. DNA restriction enzymes, T4 DNA ligase, Taq DNA polymerase, and SUMO protease were purchased from Invitrogen (Carlsbad, CA, USA).

### 4.2. Synthesis of Hybrid Peptide

Hybrid antibacterial peptide C–L derived from selected parental peptides (C and L) was designed using APD2 by fully considering its structure–activity relationship [2]. C–L was synthesized by 9-fluorenylmethoxycarbonyl solid-phase synthesis chemistry and purified by a reverse-phase semi-preparative HPLC (SBS, Shenzhen, China), and then stored at −80 °C until use.

### 4.3. PCR Amplification and Construction of Expression Vectors

The C–L gene with appropriate codons for *E. coli* (http://www.kazusa.or.jp/codon/) was synthesized (Invitrogen, USA) and cloned into pMD18-T (TaKaRa, Otsu, Japan). Plasmid DNA was isolated using a TIAN prep Midi Plasmid Kit (Tiangen, Beijing, China). The gene of C–L was amplified using Taq DNA polymerase (TaKaRa), primer C-L-F (5′-GCCGATGAAATGGAAACTGT-3′) and C-L-R (5′-GCGTACTCACTATTAGTTCCTCAG-3′). The PCR condition was 94 °C for 5 min for hot start, followed by denaturing at 94 °C for 30 s, annealing at 54 °C for 30 s, extension at 72 °C for 45 s, and finally, incubation at 72 °C for 10 min. The PCR products were separated by 2% gel electrophoresis, purified with DNA gel extraction kit (Tiangen), and inserted into the linearized pET-SUMO plasmid (Invitrogen) by TA cloning using T4 DNA Ligase (TaKaRa). The ligation mixture was transformed into *E. coli* Mach1TM-T1R cells, and the recombinant plasmid was verified by PCR amplification and sequencing.

### 4.4. Expression of SUMO–C–L Fusion Protein

The pET-SUMO–C–L plasmid was transformed into competent *E. coli* BL21 (DE3). The recombinant expression strain was cultivated in LB broth containing 50 μg/mL kanamycin at 37 °C with shaking (200 rpm) to an optical density (OD_600_) of 0.6~0.8. Isopropyl β-d-1-thiogalactopyranoside (IPTG) (1.5 mM) was then added to induce the expression of the recombinant protein at 37 °C for 5 h. The cells were harvested by centrifuging at 13,000× *g* for 5 min, and resuspended in lysis buffer (50 mM K_3_PO_4_, 400 mM NaCl, 100 mM KCl, 10% glycerol, 0.5% Triton X-100, 10 mM imidazole, pH 7.8), and disrupted by sonication at 200 W for 80 cycles (2 s working, 8 s free). After centrifuge at 13,000× *g* for 5 min at 4 °C, the supernatant was collected for tricine-SDS-PAGE analysis.

### 4.5. Purification of SUMO–C–L Fusion Protein

The supernatant was purified with a 10 mL Ni-NTA Sepharose column (GE Healthcare, Piscataway, NJ, USA). The column was pre-equilibrated with binding buffer (50 mM Na_3_PO_4_, 500 mM NaCl, pH 7.4) and the bound protein was eluted by a linear gradient of imidazole from 10 to 400 mM in binding buffer at 2 mL/min. The eluted fractions were analyzed by tricine-SDS-PAGE, and dialyzed overnight at 4 °C against 100 mM NaCl. And the fusion protein content was quantified by bicinchoninic acid (BCA) method with three replicates [37].

### 4.6. Cleavage of SUMO–C–L Fusion Protein and Purification the Recombinant C–L

The SUMO–C–L protein was cleaved by SUMO protease (Genecopoeia, Rockville, MD, USA) at 30 °C for 3 h. The recombinant C–L protein was purified with Ni-NTA Sepharose column. The enzymatic solution and dialyzed fractions were analyzed by tricine-SDS-PAGE.

### 4.7. Antibacterial Studies

Antibacterial activity of recombinant C–L was assayed by the agar diffusion method with *S. aureus* ATCC25923 as indicator strain. A dilution of the strain was spread on MH plates, cylinders were placed on the agar surface, and 100 μL of purified recombinant SUMO–C–L (100 μg/mL) and C–L (20 μg/mL) was added to each cylinder. The same volume of PBS was used as negative control. The inhibition zone was measured after incubation overnight at 37 °C. Additionally, the minimal inhibitory concentrations (MICs) of C, L, and C–L were also determined according to the Clinical and Laboratory Standards Institute (CLSI) guidelines using *S. aureus* ATCC 25923, *E. coli* CVCC245, and *L. mono.* CVCC1599 as indicator strains. The indicator strains were cultured at 37 °C to logarithmic stage and diluted to the concentration of 1 × 10^6^ cfu/mL with Mueller–Hinton (MH) broth medium, and 180 µL culture was dispensed into per well of 96-well microtiter plate. Peptides were then serially diluted, and dilutions were added into the wells (final volume 200 µL). Each assay was performed in triplicate. After incubation at 37 °C for 12 h, the plate was assessed by measuring the OD_600_. The MIC was defined as the lowest concentration (at which there was no change in optical density) required to prevent the growth of bacteria [2].

### 4.8. Hemolytic Asssay

Peptide concentrations that caused 50% hemolysis of sheep erythrocytes at 540 nm were measured to evaluate the hemolytic activities [2]. Sheep erythrocytes with 10 mM phosphate buffered saline (PBS)/0.1% (*v*/*v*) Triton X-100 (Sigma, San Francisco, CA, USA) added were used as negative/positive control. Assays were performed in triplicate.

### 4.9. Assessment of Stability

The stability of the purified recombinant C–L and synthesized C–L at different pH values (2.0–11.0) and different temperatures (37, 50, 60, 70, 80, 90, and 100 °C for 20 min) was evaluated. The susceptibility of C–L to pepsin, trypsin, and proteinase K (10 μg/mL in PBS) was measured by adding it to a solution of the peptide in PBS (1 μM) [38]. Stability of recombinant C–L and synthesized C–L was assessed by the agar diffusion method as described above and the inhibition zones were recorded. *S. aureus* ATCC25923 was used as the indicator strain and the inhibition zones were recorded.

## 5. Conclusions

In summary, an effective approach was created for overproducing a novel hybrid peptide C–L in *E. coli*. SUMO fusion technology was successfully used to eliminate the toxicity and proteolytic degradation of C–L peptide. High recovery yield of recombinant C–L with potential antimicrobial activity was obtained after efficient cleavage of SUMO protease and simple purification. The purified C–L displayed identical antibacterial and hemolytic activities to synthesized C–L, which is significantly superior to the parental peptides. Antimicrobial activity was displayed at the temperature and pH ranges commonly found in the farm animal body. In addition, C–L was, to some extent, resistant to proteolytic digestion of several common enzymes. These findings indicate that the *E. coli* expression system and SUMO fusion strategy have great potential for high-level expression of various kinds of valuable peptides and proteins. Furthermore, as a novel hybrid peptide with excellent properties, C–L is expected to serve as a useful antimicrobial or even therapeutic agent for animals or possibly humans.

## Figures and Tables

**Table 1:** The minimal inhibitory concentrations (MICs) and hemolytic activity (HC_50_) of C, L, recombinant C–L, and synthesized C–L.

Indicated Strains	MIC (μg/mL)				*p*-Value
	C	L	Recombinant C–L	Synthesized C–L	
*E. coli* CVCC 245	4.2 ± 0.35 ^c^	85.2 ± 2.00 ^a^	7.2 ± 0.14 ^b^	7.0 ± 0.21 ^b^	<0.01
*S. aureus* ATCC 25923	198 ± 10.1 ^a^	58.0 ± 2.56 ^b^	2.2 ± 0.05 ^c^	2.0 ± 0.02 ^c^	<0.01
*L. mono.* CVCC 1599	64.5 ± 3.20 ^a^	13.4 ± 0.50 ^b^	2.1 ± 0.03 ^c^	2.2 ± 0.05 ^c^	<0.01
**Indicated cell**	**HC_50_ (μg/mL)**				***p*-Value**
	C	**L**	**Recombinant C–L**	**Synthesized C–L**	
Sheep erythrocyte cell	169 ± 8.20 ^b^	32 ± 1.20 ^c^	221 ± 3.45 ^a^	219 ± 2.98 ^a^	<0.01

C, cecropin A; L, LL37; MICs, minimal inhibitory concentrations; *E. coli*, *Escherichia coli*; *S. aureus*, *Staphylococcus aureus*; *L. mono.*, *Listeria monocytogenes*; ^a,b,c^ Means with different superscripts within the same row differ (*p* < 0.01).
